# Supplementary material for: Pronounced proliferation of non-beta cells in response to beta-cell mitogens in isolated human islets of Langerhans
Source: Sci Rep. 2021 May 28;11:11283. doi: 10.1038/s41598-021-90643-3 (PMC8163757; doi:10.1038/s41598-021-90643-3)
Supplement: Supplementary file 1 — Supplementary Information. [file 41598_2021_90643_MOESM1_ESM.pdf]

Pronounced proliferation of non-β cells in response to β-cell mitogens in isolated human islets of Langerhans

Hasna Maachi, Julien Ghislain, Caroline Tremblay, Vincent Poitout

Supplementary Figure S1. Analysis of apoptosis in intact human islets. Intact human islets (N = 1) were exposed to 2.8 mM glucose (Vehicle or 2.8 Glu), harmine (10 μM), 16.7 mM glucose or HB-EGF (100 ng/ml) for 72 h. EdU (10 μM) was added throughout. Proliferation was assessed by EdU staining and apoptosis using the terminal deoxynucleotidyl transferase (TdT) FragEL DNA fragmentation assay. Representative images of FragEL (green), EdU (red) and nuclei (blue) staining for each condition are shown. Arrows highlight rare EdU^+/$FragEL^+$ nuclei. Scale bar, 50 μm. Images were acquired using an Axio Imager with ZEN 2012 software (https://www.zeiss.com/microscopy/int/products/microscope-software/zen.html).

Supplementary Figure S2. Analysis of total and proliferating C-peptide- and Nkx6.1-positive cells in intact human islets. Intact human islets (N = 3) were exposed to 2.8 mM glucose (Vehicle or 2.8 Glu), harmine (10 μM, Har), 16.7 mM glucose (16.7 Glu) and HB-EGF (100 ng/ml, HB) for 72 h. EdU (10 μM) was added throughout. C-peptide (CPEP) and Nkx6.1 were used to mark β cells and EdU for proliferation. Representative images of CPEP (green), Nkx6.1 (red) and EdU (blue) staining are shown (a). Arrows and arrowheads highlight EdU^+/$CPEP^+$/$Nkx6.1^-$ and EdU^+/ $CPEP^+$/$Nkx6.1^+$ cells, respectively. Scale bar, 50 μm. Images were acquired using an Axio Imager with ZEN 2012 software (https://www.zeiss.com/microscopy/int/products/microscope-software/zen.html). A minimum of 1,500 $CPEP^+$ cells were counted for each sample. The ratio of

total (b) and proliferating (c) Nkx6.1$^+$/CPEP$^+$ cells for each condition are shown. Graphs were generated using GraphPad Prism 9 software (https://www.graphpad.com/scientific-software/prism/).

Supplementary Figure S3. Analysis of CK19-positive cells in intact human islets. Intact human islets (N = 2) were stained for C-peptide (CPEP, green), cytokeratin-19 (CK19, red) and nuclei (blue). Images of islet sections from 2 donors are shown. Scale bar, 50 µm. Images were acquired using an Axio Imager with ZEN 2012 software (https://www.zeiss.com/microscopy/int/products/microscope-software/zen.html).

Supplementary Figure S4. Analysis of cell proliferation in intact human islets by flow cytometry. Intact human islets were exposed to 2.8 mM glucose, harmine (10 µM), 16.7 mM glucose and HB-EGF (100 ng/ml) for 72 h. EdU (10 µM) was added throughout. At the end of the treatment islets were dispersed into single cells and analyzed by flow cytometry for C-peptide (CPEP), glucagon (GCG) and EdU (a-c & e-g) or cytokeratin 19 (CK19) and EdU (d & h). Representative plots showing gating used for selection of islet cells (a), live cells (a), CPEP$^+$, GCG$^+$, CPEP$^-$/GCG$^-$ (c) and CK19$^+$ (d) cells and proliferating EdU$^+$/GCG$^+$ (e), EdU$^+$/CPEP$^+$ (f), EdU$^+$/CPEP$^-$/GCG$^-$ (g) and EdU$^+$/CK19$^+$ (h) cells. Plots were generated using FlowJo v10.7 software (https://www.flowjo.com/solutions/flowjo).

Supplementary Figure S5. Comparison of the percentage of proliferating cells detected using different approaches. (a-e) Intact human islets were exposed to 2.8 or 16.7 mM glucose, harmine (10 µM) or HB-EGF (100 ng/ml) for 72 h. EdU (10 µM) was added throughout. Proliferation was assessed in parallel for each donor by immunohistochemistry (IHC) (a-e) or flow cytometry (FC) (c-e) and presented as a percentage of EdU$^+$/CPEP$^+$ over total CPEP$^+$ (a-c), EdU$^+$/Nkx6.1$^+$ over

total Nkx6.1$^+$ (a), Ki67$^+$/CPEP$^+$ over total CPEP$^+$ (b), EdU$^+$/GCG$^+$ over total GCG$^+$ (d) and EdU$^+$/CK19$^+$ over total CK19$^+$ (e). Scatterplots of all treatment conditions combined are presented to illustrate the correlation between β-cell proliferation assessed by IHC for CPEP versus Nkx6.1 (6 donors) (a) and Ki67 versus EdU (3 donors) (b) and proliferation of CPEP$^+$ (5 donors) (c), GCG$^+$ (4 donors) (d) and CK19$^+$ (4 donors) (e) cells assessed by IHC versus FC. These data are presented in the main figures 2 and 3 (a), 3 and 4 (b) and 7 (c-e). Graphs were generated using GraphPad Prism 9 software (https://www.graphpad.com/scientific-software/prism/). Significance was tested using two-tailed paired t-tests. $P<0.05$ was considered significant. NS, not significant. R, Pearson correlation coefficient.

Supplementary table S1.xlsx

Supplementary table S2.docx

Supplementary Figure S1

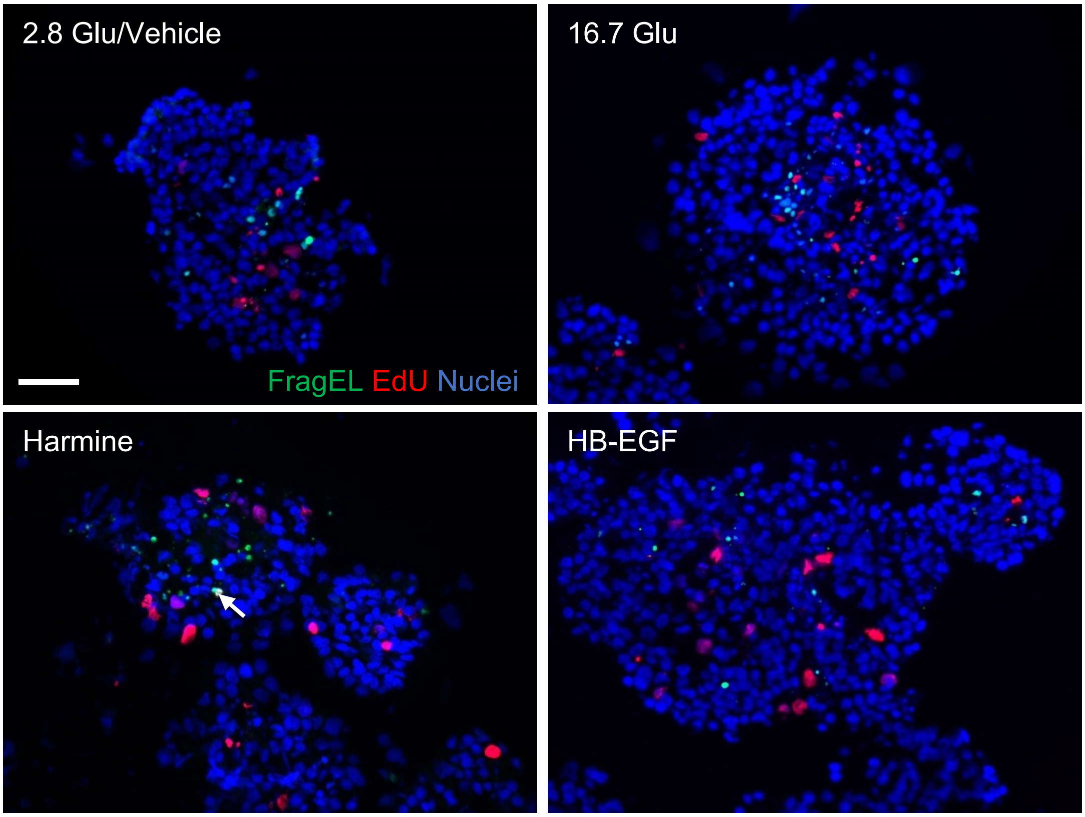

Supplementary Figure S2

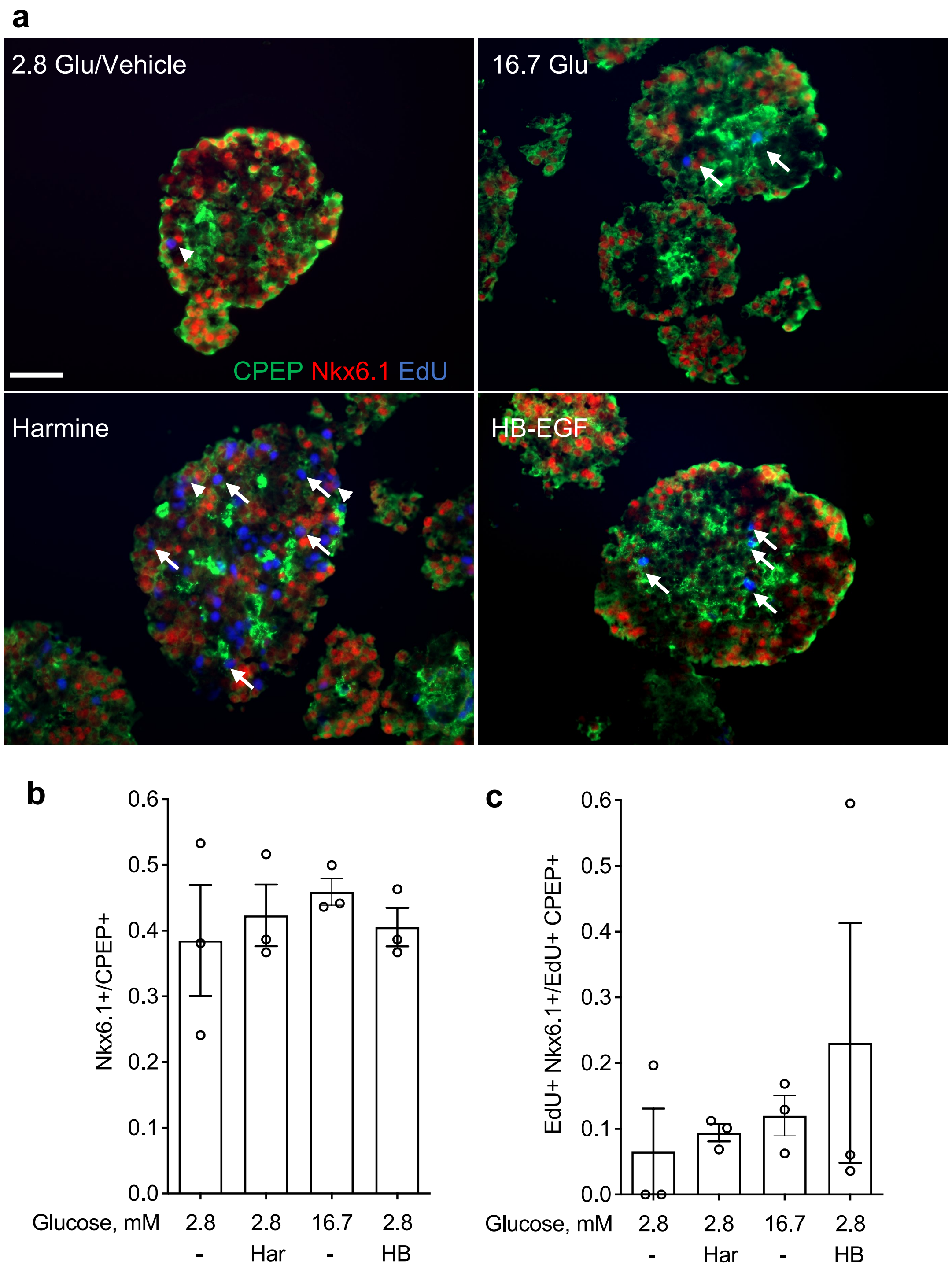

Supplementary Figure S3

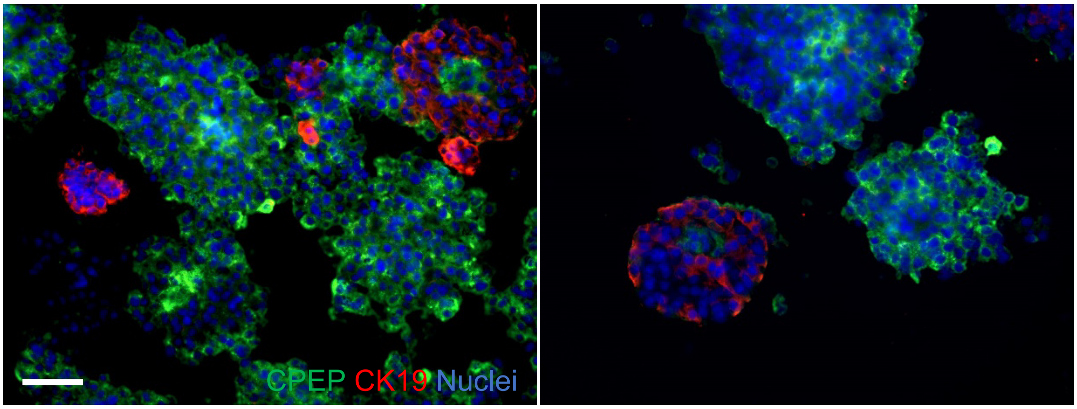

Supplementary Figure S4

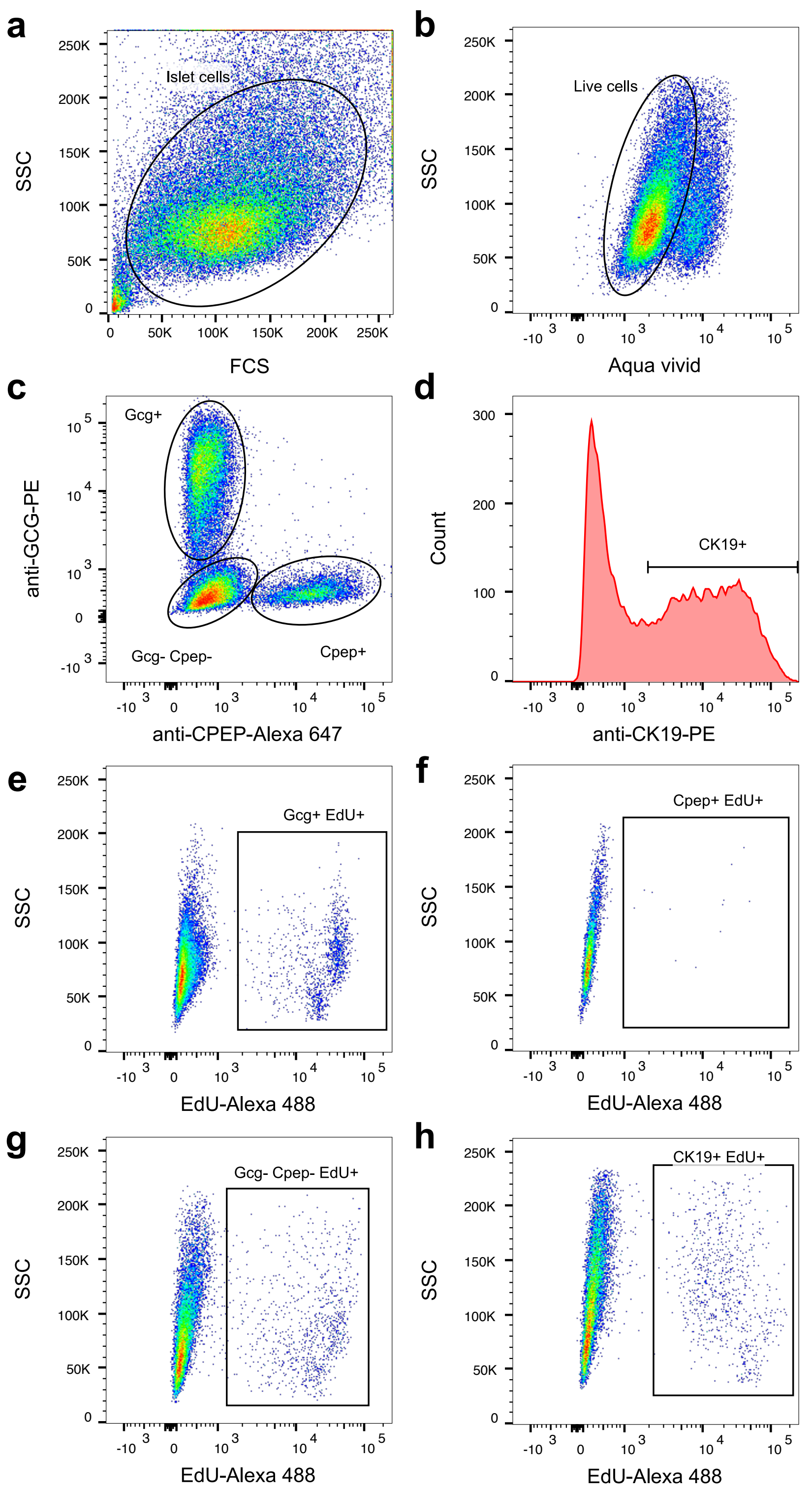

Supplementary Figure S5

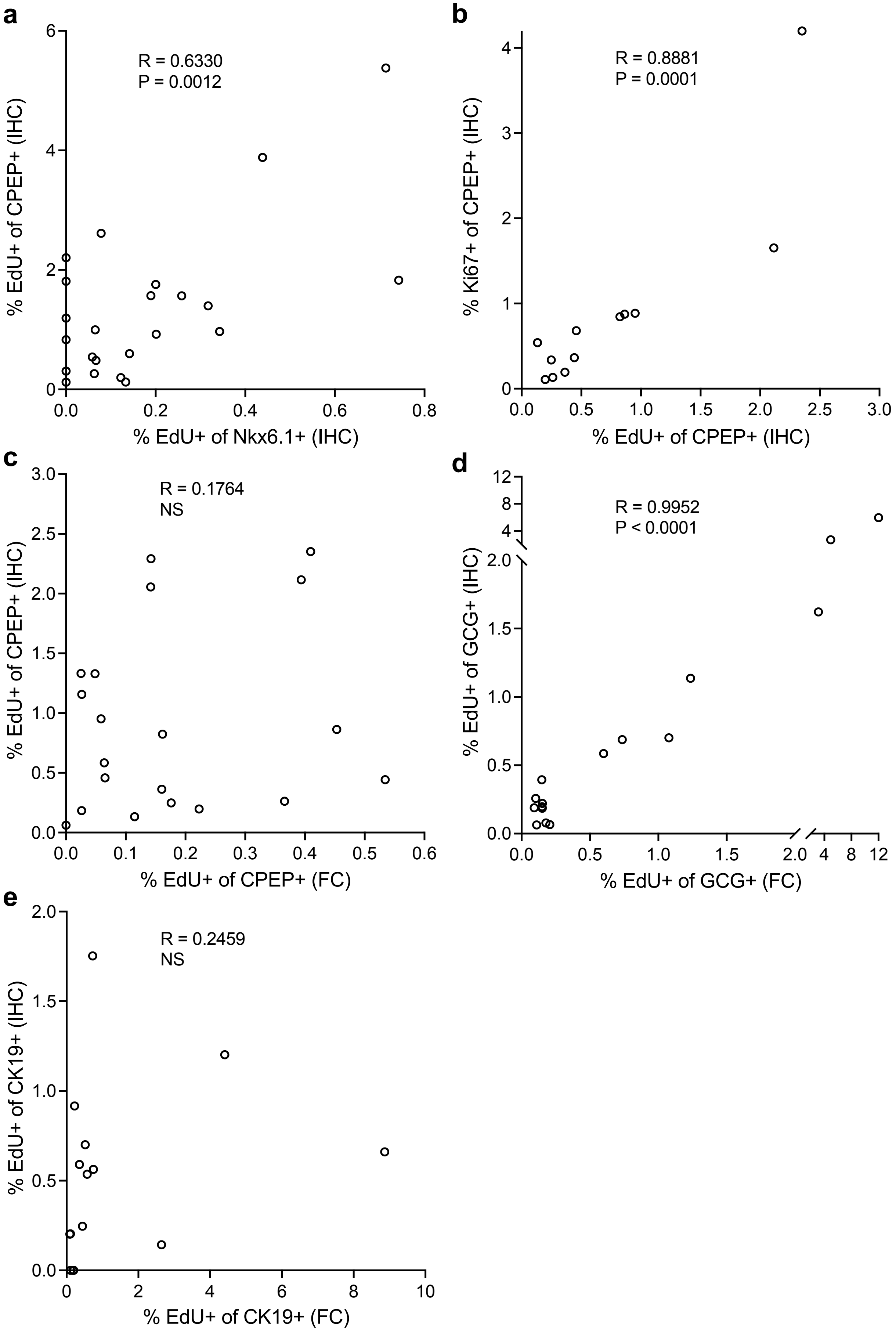

Supplementary Table S1. Human islet donors used in study.									
**Donor**	**1**	**2**	**3**	**4**	**5**	**6**	**7**	**8**	**9**
**Unique identifier**	R340	SAMN12924398	SAMN13027809	R346	SAMN13134368	R348	R341	R344	SAMN11791244
**Donor Age (years)**	36	38	43	27	62	43	42	59	47
**Donor Sex (M/F)**	M	M	F	M	M	F	M	M	M
**Donor BMI (kg/m^2)**	23.3	28.0	31.6	24.1	31.3	16.4	30	27.8	36.1
**Donor HbA1c**	5.3	5.4	5.7	5.7	5.5	5.7	Not Reported	5.5	5.7
**Origin/source of islets**	Alberta IsletCore	IIDP	IIDP	Alberta IsletCore	IIDP	Alberta IsletCore	Alberta IsletCore	Alberta IsletCore	IIDP
**Islet isolation center**	Alberta IsletCore	The Scharp-Lacy Research Institute	The Scharp-Lacy Research Institute	Alberta IsletCore	The Scharp-Lacy Research Institute	Alberta IsletCore	Alberta IsletCore	Alberta IsletCore	Southern California Islet Cell Resource Center
**Donor history of diabetes**	No	No	No	No	No	No	No	No	No
**Cause of death**	NDD-Neurological	Head trauma	Anoxia	NDD-Neurological	Cerebrovascular/stroke	NDD-Neurological	NDD-Neurological	NDD-Neurological	Cerebrovascular/stroke
**Warm ischemia time (h)**	Not Reported	Not Reported	0.2	Not Reported	Not Reported	Not Reported	Not Reported	Not Reported	0.3
**Cold ischemia time (h)**	12.5	8.8	11.1	12	10.2	15.5	15.75	11.5	6.4
**Estimated purity (%)**	95	80	90	90	90	90	95	95	95
**Estimated viability (%)**		95	95		95				96
**Glucose-stimulated insulin secretion stimulation index (SI)**	SI (G2.8mM-G16.7mM)= 4.56	SI (G2.8mM-G28mM)= 1.8	SI (G2.8mM-G28mM)= 2.8	SI (G2.8mM-G16.7mM)= 6.17	Not Reported	SI (G2.8mM-G16.7mM)= 1.63	SI (G2.8mM-G16.7mM)= 9.81	SI (G2.8mM-G16.7mM)= 3.11	SI (G2.8mM-G28mM)= 2.5
**FACS data**									
**Single cells/total cells (%)**									
**Live cells/single cells (%)**									
**CPEP+ cells/live cells (%)**									
**GCG+ cells/live cells (%)**									
**CPEP-, GCG- cells/live cells (%)**									

**Donor**	**10**	**11**	**12**	**13**	**14**	**15**	**16**	**17**	**18**
**Unique identifier**	R301	SAMN10869303	SAMN10023853	SAMN09862214	SAMN09370567	SAMN10410585	H2182	SAMN09929594	SAMN10516338
**Donor Age (years)**	18.0	25.0	25.0	31.0	32.0	38.0	48.0	51.0	52.0
**Donor Sex (M/F)**	M	M	F	M	M	M	F	F	F
**Donor BMI (kg/m^2)**	19	27.7	33.5	31.8	28.5	28.2	38.5	29.7	26.9
**Donor HbA1c**	5.0	5.2	5.1	5.3	5.2	5.9		5.8	4.5
**Origin/source of islets**	Alberta IsletCore	IIDP	IIDP	IIDP	IIDP	IIDP	Alberta islet lab	IIDP	IIDP
**Islet isolation center**	Alberta IsletCore	The Scharp-Lacy Research Institute	Southern California Islet Cell Resource Center	Southern California Islet Cell Resource Center	University of Wisconsin	The Scharp-Lacy Research Institute	Alberta islet lab	Southern California Islet Cell Resource Center	The Scharp-Lacy Research Institute
**Donor history of diabetes**	No	No	No	No	No	No	No	No	No
**Cause of death**	NDD-Neurological	Anoxia	Anoxia	Head trauma	Head trauma	Head trauma		Cerebrovascular/stroke	Cerebrovascular/stroke
**Warm ischemia time (h)**	Not Reported	0.5	0.1	Not Reported	0.1	Not Reported	Not Reported	Not Reported	Not Reported
**Cold ischemia time (h)**	5	8.4	7.0	5.5	11.5	7.3		6.9	6.3
**Estimated purity (%)**	75	90	75	80	95	90		75	80
**Estimated viability (%)**		95	96	95	91	95		94	95
**Glucose-stimulated insulin secretion stimulation index (SI)**	SI (G2.8mM-G16.7mM)= 9.21	SI (G2.8mM-G28mM)= 8.9	SI (G2.8mM-G28mM)= 0.7	SI (G2.8mM-G28mM)= 2.0	SI (G2.8mM-G28mM)= 1.8	SI (G2.8mM-G28mM)= 6.6	Not Reported	SI (G2.8mM-G28mM)= 2.1	SI (G2.8mM-G28mM)= 6.5
**FACS data**									
**Single cells/total cells (%)**	87.6	89.6	85.0	93.9	95.6	93.2	89.6	96.1	92.5
**Live cells/single cells (%)**	88.9	86.5	85.2	83.2	88.5	74.7	77.9	91.1	83.5
**CPEP+ cells/live cells (%)**	8.6	43.2	23.4	3.1	6.7	19.3	19.3	9.2	13.7
**GCG+ cells/live cells (%)**	6.9	35.8	18.3	28.9	62.1	29.9	12.9	55.6	44.2
**CPEP-, GCG- cells/live cells (%)**	84.5	21.0	58.3	68.0	31.2	50.8	67.8	35.2	42.1

**Donor**	**19**	**20**	**21**	**22**	**23**	**24**	**25**	**26**	**27**
**Unique identifier**	R305	R271	SAMN09228907	R356	HP20071-01	SAMN15337453	R365	H2330	SAMN16081314
**Donor Age (years)**	60.0	60.0	62	45	42	26	31	49	24
**Donor Sex (M/F)**	M	F	M	F	M	M	F	F	M
**Donor BMI (kg/m^2)**	21	25.9	36.1	29.7	27.97	24.2	20.3	27.2	20.1
**Donor HbA1c**	5.6	5.5	5.8	5.1	4.9		4.8		5.4
**Origin/source of islets**	Alberta IsletCore	Alberta IsletCore	IIDP	Alberta IsletCore	Prodo labs	IIDP	Alberta IsletCore	University of Alberta	IIDP
**Islet isolation center**	Alberta IsletCore	Alberta IsletCore	The Scharp-Lacy Research Institute	Alberta IsletCore	Prodo labs	The Scharp-Lacy Research Institute	Alberta IsletCore	Alberta islet distribution program	The Scharp-Lacy Research Institute
**Donor history of diabetes**	No	No	No	No	No	No	No	No	No
**Cause of death**	NDD-Neurological	NDD-Neurological	Anoxia	NDD-Neurological	Head trauma	Head trauma	NDD-Neurological	Not Reported	Head trauma
**Warm ischemia time (h)**	Not Reported	Not Reported	Not Reported	Not Reported	Not Reported	Not Reported	Not Reported	Not Reported	Not Reported
**Cold ischemia time (h)**	10	13.5	10.7	14	Not Reported	10.2	6.75		7.8
**Estimated purity (%)**	80	95	90	80	Not Reported	98	95	72.5	95
**Estimated viability (%)**			95			95		85.5	95
**Glucose-stimulated insulin secretion stimulation index (SI)**	SI (G2.8mM-G16.7mM)= 1.29	SI (G2.8mM-G16.7mM)= 4.98	SI (G2.8mM-G28mM)= 3.9	SI (G2.8mM-G16.7mM)= 5.49	Not Reported	SI (G2.8mM-G28mM)= 4.4	SI (G2.8mM-G16.7mM)= 5.34	Not Reported	Not Reported
**FACS data**									
**Single cells/total cells (%)**	91.4	98.6	92.6						88.4
**Live cells/single cells (%)**	85.5	91.7	87.6						84.3
**CPEP+ cells/live cells (%)**	31.1	34.2	2.6						17.8
**GCG+ cells/live cells (%)**	14.2	37.0	83.1						56.8
**CPEP-, GCG- cells/live cells (%)**	54.8	28.8	14.4						25.5

**Donor**	**28**	**29**	**30**	**31**	**32**	**33**	**34**	**35**
**Unique identifier**	SAMN16114998	R391	SAMN16871526	SAMN17277513	SAMN17528599	SAMN17928660	SAMN18021384	SAMN18092805
**Donor Age (years)**	24	67	62	43	60	62	56	56
**Donor Sex (M/F)**	F	M	M	F	M	M	M	M
**Donor BMI (kg/m^2)**	45.5	24.5	21.0	36.5	29.9	25.9	24.2	21.6
**Donor HbA1c**	5.2	4.9	5.3	5.2	5.8	5.1	5.8	5.1
**Origin/source of islets**	IIDP	Alberta IsletCore	IIDP	IIDP	IIDP	IIDP	IIDP	IIDP
**Islet isolation center**	University of Wisconsin	Alberta IsletCore	The Scharp-Lacy Research Institute	Southern California Islet Cell Resource Center	University of Pennsylvania	Southern California Islet Cell Resource Center	The Scharp-Lacy Research Institute	Southern California Islet Cell Resource Center
**Donor history of diabetes**	No	No	No	No	No	No	No	No
**Cause of death**	Anoxia	Not Reported	Cerebrovascular/stroke	Cerebrovascular/stroke	Anoxia	Head trauma	Anoxia	Cerebrovascular/stroke
**Warm ischemia time (h)**	Unknown	Not Reported	Not Reported	0.4	Not Reported	Not Reported	Unknown	Not Reported
**Cold ischemia time (h)**	4.6	17	9.7	5.3	7.7	7.4	6.7	5.8
**Estimated purity (%)**	85	95	90	90	85	90	90	90
**Estimated viability (%)**	98		95	96	98	96	95	96
**Glucose-stimulated insulin secretion stimulation index (SI)**	Not Reported	SI (G2.8mM-G16.7mM)=2.31	SI (G2.8mM-G28mM)= 1.1	SI (G2.8mM-G28mM)= 2.7	SI (G2.8mM-G28mM)= 1.3	Not Reported	Not Reported	Not Reported
**FACS data**								
**Single cells/total cells (%)**	89.3	91.4	91.6	86.4	86.5	92.9		
**Live cells/single cells (%)**	83.4	94.5	93.6	90.5	91.0	90.4		
**CPEP+ cells/live cells (%)**	16.6	41.6	15.9	16.5	32.9	12.6		
**GCG+ cells/live cells (%)**	54.7	31.3	72.2	46.8	49.3	67.7		
**CPEP-, GCG- cells/live cells (%)**	28.7	27.1	11.8	36.6	17.8	19.6		

Supplementary Table S2 Source of antibodies used in study.

Antibody	Dilution/Concentration	Company	Cat #
**Immunohistochemistry/ Immunocytochemistry**			
Rabbit polyclonal Anti-Nkx6.1	1:100	Novus	NBP1-82553
Rat monoclonal Anti-C-Peptide	5 μg/ml	DSHB	GN-ID4
Mouse monoclonal Anti-Cytokeratin 19	1:500	Abcam	Ab9220
Mouse monoclonal Anti-Glucagon	1:400	Abcam	Ab10988
Rabbit polyclonal Anti-Ki67	1:400	Abcam	Ab15580
**Flow cytometry**			
Alexa Fluor® 647 Mouse Anti-C-Peptide	1:50	BD Biosciences	565831
PE Mouse Anti-Glucagon	1:50	BD Biosciences	565860
Mouse monoclonal Anti-Cytokeratin 19	1:500	Abcam	Ab9220
